# Self-reported knowledge on diabetes and its related factors among Chinese college students: a cross-sectional study

**DOI:** 10.1136/bmjopen-2016-011963

**Published:** 2016-09-08

**Authors:** Ying Xu, Dongdong Zhang, Kaiqian Liu, Yanfang Guo, Yi Yang

**Affiliations:** 1 Department of Chronic non-communicable disease prevention and control, Baoan Chronic Diseases Prevent and Cure Hospital, Shenzhen, China; 2 Department of Epidemiology and Biostatistics, School of Public Health, Guangdong Pharmaceutical University, Guangzhou, China

**Keywords:** EPIDEMIOLOGY, PREVENTIVE MEDICINE, PUBLIC HEALTH

## Abstract

**Objectives:**

An increasing trend in the prevalence of type 2 diabetes has been observed among youths; however, little is known about how informed young people are of its existence and dangers. This study is to assess the level of knowledge on type 2 diabetes among Chinese college students and to explore related factors influencing the knowledge.

**Setting:**

A cross-sectional survey was conducted among college students in Guangzhou, China, from September to November 2013.

**Participants:**

A total of 658 students were randomly recruited using a multistage sampling method and were invited to participate in the confidential interviews.

**Primary and secondary outcome measures:**

Self-reported knowledge on diabetes and its main sources were measured by a self-designed questionnaire.

**Results:**

A total of 521 students participated in this study. The mean total score of knowledge was 13.3±3.44 of 22. Less than 50% of participants could correctly answer the questions about the onset of type 2 diabetes, the adverse effects of sedentary lifestyles, the complications, the therapeutic methods and the monitoring index of diabetes. The factors associated with higher levels of knowledge about type 2 diabetes in stepwise regression models were: being in a high grade, having a better academic performance, having a medical specialty and having relatives or friends with diabetes. Newspapers and books (61.4%), television and the Internet (46.3%) were the major sources of knowledge about type 2 diabetes, and more than half of the participants (55.9%) considered that medical staff was the most reliable source.

**Conclusions:**

The college students had limited knowledge about type 2 diabetes. Public education, especially among individuals with non-medical specialties, a low-level grade, poor academic performance or no relatives and friends with diabetes, would be extremely beneficial.

**Strengths and limitations of this study**

- To the best of our knowledge, this is one of the first studies to systematically assess the level of knowledge on type 2 diabetes from five respects and the potential-related factors influencing the knowledge among Chinese college students.
- The results can provide evidence in developing health education programmes for young adults to face the challenge of increasing trend of diabetes to be younger.
- The level of knowledge might be overestimated due to the nature of a higher rate of rejection and self-reported data.

## Introduction

Diabetes is a major, non-communicable disease with increasing prevalence globally^1^ and will be the seventh leading cause of death worldwide by 2030 as estimated by the WHO report in 2011.^2^ Type 2 diabetes affects the most productive midlife period but has also started to appear in younger age groups.^3^ Type 2 diabetes is now increasingly diagnosed among adolescents and young adults, but it is a potentially preventable disease through a combination of lifestyle modification and pharmaceutical treatment.^4–6^ Adopting a healthy lifestyle, including healthy diet choices and exercise habits, plays a critical role in the prevention and control of type 2 diabetes and has been associated with lower risk of type 2 diabetes.^1^ ^7^ ^8^ Knowledge forms a basis for the adoption of good health-related practices and these lifestyle changes at the time of youth facilitate maintenance throughout a person's life.^9^ ^10^ Hence, it is crucial that young people could be well informed about the preventive measures of type 2 diabetes, its risk factors, complications and therapeutic methods.

However, except for some studies conducted among medical students,^11–13^ Scare studies have evaluated the diabetic knowledge among college students. A study conducted among university students in Ajman in the United Arab Emirates have shown that the participants' level of type 2 diabetes-related knowledge, especially the fact that type 2 diabetes can be prevented or delayed, was not adequate^10^ Another study, which included college students in public schools in Morelos, Central Mexico, also showed that the participants had low levels of knowledge about type 2 diabetes diagnoses and prevention.^14^ Given the differences in socioeconomics and culture across different countries, the purpose of this study was to assess type 2 diabetes-related knowledge, including general knowledge, risk factors, clinical-related knowledge and some potential factors influencing this knowledge among Chinese college students. The results of this study, by identifying areas of knowledge deficiency and some influencing factors, can assist in developing health education programmes for young adults.

## Materials and methods

### Participants

The cross-sectional study was conducted among college students in Guangzhou, China, from September to November 2013. The participants were recruited by multistage sampling methods. First, 8 of 16 colleges were randomly sampled. Second, three classes were sampled from each of the eight colleges. Third, about 30 students were sampled in every selected class. Finally, a total of 658 college students enrolled in confidential interviews. Of them, 79 refused to participate in this study due to lack of interest and 579 returned their questionnaires to the trained investigators, with 521 of 579 supposedly valid ones (valid questionnaire referred to missing items less than four ones). According to the target significance level of 0.05, a tested rate of awareness of 45% (referred to the reported rate 45.81% in Chinese adults (17)), a sample size of 500 achieves 99.5% power to detect a difference of 0.10 using a two-sided binomial test.

### Data collection

A structured questionnaire with closed-ended questions was developed after an extensive literature search and consulting experts. The questionnaire was pretested with a group of 15 college students to identify any problems related to question design, flow or interpretation. Following that identified inconsistencies and inaccuracies were corrected. The reliability of the questionnaire was assessed by Cronbach's α coefficients with a value of 0.686 in this study.

After the investigators introduced the objective of the study and ways to fill in the questionnaires in detail, participants were required to answer the following questions using anonymous self-administered methods: (1)general characteristics (see table 1), including sex, specialty, grade, academic performance self-assessment, the habit of eating breakfast, sleep time and frequency of taking physical activity, self-reported height and weight, and having relatives and/or friends with diabetes or not; (2) knowledge about type 2 diabetes, evaluated by 22 items (see table 2) including general knowledge (four items), risk factors (five items), symptoms (four items), complications (five items) and diagnosis and treatment (four items); and (3) sources of knowledge and the authoritative sources that they considered.

**Table 1:** General characteristics and their associations with scores in different areas of diabetic knowledge among the college students (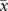
±s)

Characteristic	Number	Per cent	General (0–4)	Risk factors (0–5)	Symptoms (0–4)	Diagnosis and treatment (0–4)	Complications (0–5)	Total score (0–22)
Gender								
Men	173	33.2	2.5±0.94	2.9±1.38	3.0±1.22	2.1±0.69	2.3±1.58	12.8±3.79
Women	348	66.8	2.7±0.84	3.1±1.28	3.2±1.07	2.2±0.72	2.3±1.47	13.5±3.23
p Value			0.037	0.08	0.07	0.20	0.63	0.023
Age								
≤20	288	55.3	2.5±0.83	2.9±1.37	3.0±1.16	2.1±0.70	2.1±1.40	12.6±3.22
>20	233	44.7	2.8±0.85	3.2±1.23	3.2±1.23	2.2±0.71	2.7±1.59	14.1±3.41
p Value			<0.001	0.020	0.001	0.27	<0.001	<0.001
Grade								
Grade 1	126	24.2	2.3±0.77	2.8±1.33	3.0±1.15	2.1±0.67	1.8±1.27	12.0±2.97
Grade 2	138	26.5	2.4±0.86	2.7±1.47	2.8±1.19	2.2±0.68	2.1±1.49	12.2±3.45
Grade 3	170	32.6	2.8±0.78	3.3±1.17	3.3±1.00	2.1±0.74	2.6±1.47	13.9±3.06
Grade 4	87	16.7	2.9±1.00	3.6±1.05	3.4±1.07	2.3±0.76	3.2±1.47	15.4±3.43
p Value			<0.001	<0.001	<0.001	0.09	<0.001	<0.001
Specialty								
Medical	228	43.8	2.8±0.89	3.2±1.25	3.4±0.94	2.2±0.72	2.8±1.54	14.4±3.25
Non-medical	293	56.2	2.7±0.84	2.9±1.35	2.9±1.20	2.1±0.71	2.0±1.38	12.4±3.33
p Value			<0.001	0.006	<0.001	0.06	<0.001	<0.001
Academic performance								
Worse	39	7.5	2.3±1.07	2.8±1.42	2.9±1.2	2.1±0.73	2.3±1.57	12.4±3.66
Good	342	65.6	2.6±0.83	3.0±1.31	3.1±1.11	2.1±0.69	2.3±1.51	13.1±3.37
Better	140	26.9	2.6±0.91	3.1±1.30	3.2±1.11	2.3±0.75	2.6±1.46	13.9±3.48
p Value			0.06	0.24	0.39	0.07	0.10	0.025
BMI								
<18.5	192	36.9	2.6±0.88	2.9±1.31	3.1±1.08	2.1±0.72	2.3±1.55	13.1±3.52
18.5–23.9	287	55.1	2.6±0.83	3.0±1.33	3.2±1.14	2.2±0.69	2.4±1.52	13.3±3.32
>23.9	15	2.9	3.0±0.85	3.3±1.33	3.2±1.15	2.5±0.74	3.2±1.01	15.2±3.12
Missing value	27	5.2	2.6±1.22	3.0±1.3	3.0±1.22	2.2±0.85	2.3±1.24	13.1±4.09
p Value			0.15	0.59	0.94	0.11	0.08	0.06
Skipping breakfast (>2 days/week)								
Yes	69	13.2	2.5±0.95	3.1±1.26	3.1±1.09	2.2±0.72	2.5±1.35	13.5±3.10
No	452	86.8	2.6±0.86	3.0±1.32	3.1±1.13	2.2±0.71	2.4±1.53	13.2±3.49
p Value			0.52	0.33	0.91	0.60	0.41	0.47
Physical activity (<3 days/week)								
Yes	311	59.7	2.6±0.82	3.0±1.31	3.1±1.13	2.1±0.72	2.4±1.51	13.2±3.34
No	210	40.3	2.6±0.95	3.0±1.32	3.2±1.11	2.2±0.69	2.4±1.50	13.3±3.60
p Value			0.43	0.95	0.78	0.043	0.98	0.78
Sleep time (<7 hours/day)								
Yes	166	31.9	2.6±0.90	2.9±1.30	3.1±1.16	2.2±0.70	2.4±1.46	13.2±3.52
No	355	68.1	2.6±0.86	3.1±1.32	3.2±1.10	2.1±0.72	2.4±1.53	13.3±3.41
p Value			0.56	0.23	0.42	0.14	0.76	0.66
Relatives or friends with diabetes								
Yes	176	33.8	2.7±0.85	3.1±1.27	3.2±1.08	2.1±0.71	2.5±1.41	13.6±3.28
No	242	46.4	2.6±0.89	3.1±1.29	3.1±1.12	2.2±0.74	2.3±1.54	13.3±3.49
Unknown	103	19.8	2.5±0.87	2.7±1.40	3.1±1.17	2.1±0.63	2.3±1.61	12.7±3.57
p Value			0.21	0.010	0.40	0.49	0.57	0.07
Overall	521		2.6±0.87	3.0±1.32	3.1±1.12	2.2±0.71	2.4±1.51	13.3±3.44

BMI, body mass index.

**Table 2:** Correct response for interview questions on type 2 diabetes, among the 521 college students

Statements	Number	Per cent
*General knowledge of type 2 diabetes*		
1. Diabetes is associated with abnormal insulin	447	85.8
2. Diabetes is a non-acute onset disease	104	20.0
3. Diabetes is preventable and controlled	434	83.3
4. Diabetes can be cured	369	70.8
*Knowledge of risk factors for type 2 diabetes*		
5. Sugar intake can increase the risk of diabetes	389	74.7
6. Greasy food intake can increase the risk of diabetes	277	53.2
7. Obesity	353	67.8
8. Sedentary lifestyles	253	48.6
9. Family history	291	55.9
*Knowledge of symptoms of type 2 diabetes*		
10. Excess feeling of thirst	427	82.0
11. Excessive eating	427	82.0
12. Excess urination	401	77.0
13. Unexplained weight loss	376	72.2
*Knowledge of diagnosis and treatment of type 2 diabetes*		
14. Urine sugar can be used as diagnostic criteria of type 2 diabetes except for blood sugar	367	70.4
15. Proper diet and exercise is the preferred treatment for type 2 diabetes	184	35.3
16. Hb1Ac is a better monitoring index of blood glucose fluctuations	62	11.9
17. What's the target of blood sugar control?	513	98.5
*Knowledge of complications of type 2 diabetes*		
18. Cardiovascular disease	361	69.3
19. Diabetic foot	280	53.7
20. Kidney problems	317	60.8
21. Eye problems	177	34.0
22. Diabetic peripheral neuropathy	99	19.0

HbA1c, glycated haemoglobin.

A scoring system was created by giving one score for each correct answer and a zero score for incorrect and uncertain answers. Questions that contained more than one correct answer were given one score for each correct answer. The total score ranged from 0 to 22.

### Statistical analysis

Categorical variables were described by frequency, and continuous variables were described by means±SD and tested by analysis of variance. Stepwise linear regression models (p in <0.05, p out >0.10) were fitted to explore the factors influencing the level of diabetic knowledge.

All of the analyses were carried out using the SPSS statistical package (V.17.0, SPSS , Chicago, Illinois, USA), and a p value <0.05 (two-tailed) was considered to be significant.

## Results

In all, 173 men (33.2%) and 348 women (66.8%) were included in the final analysis. Among them, 228 (43.8%) were medical students and 293 (56.2%) were non-medical students. The number of students was 126, 138, 170 and 87 from college grades 1 to 4, respectively. More than 50% of the students were in the normal range of body mass index (BMI) (18.5–23.9 kg/m^2^) and only 2.9% were overweight and obese (BMI>24 kg/m2^15^). Almost 60% reported taking physical activity <3 days/week. About one-third reported that either their relatives or their friends had been diagnosed with diabetes (see table 1).

Fewer students reported that they knew that diabetes is a non-acute onset disease (20.0%) and glycated haemoglobin is a better monitoring index of blood glucose fluctuations (11.9%). Over one-third reported that proper diet and exercise is the preferred treatment for diabetes (35.3%). Nearly one-third and one-fifth reported that eye problems (34.0%) and diabetic peripheral neuropathy (19.0%) are common diabetes-related complications. Half of the students thought that a sedentary lifestyle is associated with the risk of diabetes (48.6%) and more than 70% of students claimed that they knew the four typical symptoms of diabetes, including an excessive feeling of thirst, excessive eating, excessive urination and unexplained weight loss (see table 2).

Regarding the score of knowledge, women had a higher score than men, especially in the area of general knowledge and total score. Compared with the students who had a poor academic performance and had no diabetic relatives and friends, the students who reported having a good or better academic performance and had diabetic relatives and/or friends had higher scores in total scores and in the area of diabetic risk factors. Furthermore, the higher grades olders had higher scores than the lower grade youngsters. As expected, medical students had higher scores in almost all fields of diabetic knowledge than non-medical students. However, we found no association of BMI and lifestyle, including inactivity lifestyle and less sleep time, with diabetic knowledge (see table 1). The stepwise multivariable linear regression analysis showed that a higher grade, medical specialty, better academic performance and having relatives or friends with diabetes were the predictors for a higher total score of diabetic knowledge (see table 3).

**Table 3:** Associations between general characteristics and scores of knowledge in multivariate stepwise linear regression analysis

	General		Risk factors		Symptoms		Diagnosis and treatment		Complications		Total score	
Characteristics	b	p	b	p	b	p	b	p	b	p	b	p
Gender	–		–		–		–		–		–	
Age	–		−0.344	0.042	0.248	0.011	–		−0.399	0.032	–	
Grade	0.207	<0.001	0.41	<0.001	–		–		0.549	<0.001	0.975	<0.001
Specialty	−0.152	0.043	–		−0.433	<0.001	−0.128	0.041	−0.614	<0.001	−1.529	<0.001
Academic performance	–		–		–		0.114	0.044	–		0.621	0.012
BMI	–		–		–		–		–		–	
Skipping breakfast (>2 days/week)	–		–		–		–		–		–	
Physical activity (<3 days/week)	–		–		–		0.161	0.011	–		–	
Sleep time (<7 hours/day)	–		–		–		–		–		–	
Relatives or friends with diabetes	−0.101	0.045	−0.216	0.006	–		–		–		−0.502	0.008

b: regression coefficient, p: p Value: not be included in the linear regression models using stepwise method (p in<0.05, p out>0.10).

The independent variables in the linear regression models were coded as the following: Gender (men=1, women=2), age (≤20=1, >20=2), grade (grade 1=1, grade 2=2, grade 3=3, grade 4=4).

Specialty (medical=1, non-medical=2), academic performance (worse=1, good=2, better=3), BMI (<18.5=1, 18.5–23.9=2, >23.9=3, missing value=4), skipping breakfast (yes=1, no=2).

Physical activity (<3 days/week=1, ≥3 days/week=2), sleep time (<7 hours/day=1, ≥7 hours/day=2), relatives or friends with diabetes (yes=1, no=2, unknown=3).

BMI, body mass index.

**Figure 1:**
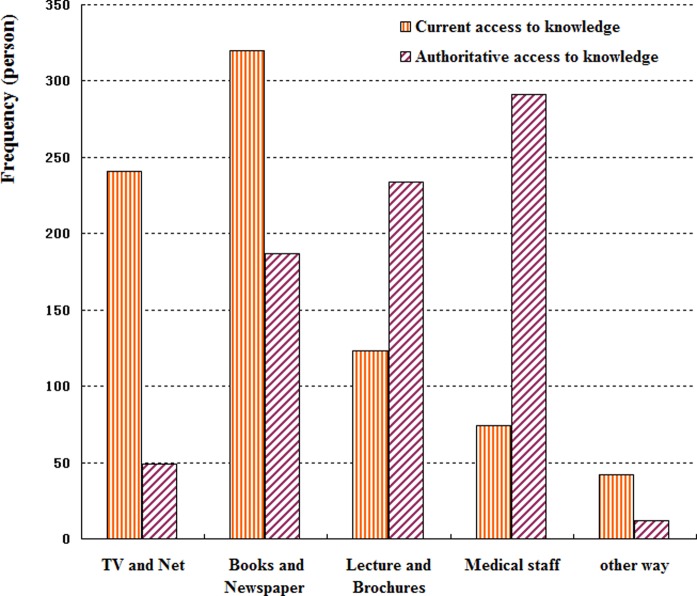
Sources of diabetic knowledge among the 521 college students. Notably, the two major access.

Notably, the two major access areas to knowledge reported by students were via books and newspapers (61.4%) and TV and the Internet (46.3%). However, most of the students considered that the most authoritative access to knowledge was from medical staff (55.9%), followed by lectures and brochures (44.9%; see figure 1).

## Discussion

This study indicated that the college students had low levels of knowledge about type 2 diabetes with the rates of correct answers being <50% in the following areas: its chronic onset, sedentary lifestyles associated with the risk of diabetes, the monitoring index of blood glucose fluctuations, healthy dietary and exercise habits for treatment and its complications with eye problems and diabetic peripheral neuropathy. In addition, a higher grade, a medical specialty, better academic performance and having relatives or friends with diabetes were significantly associated with higher scores of knowledge regarding diabetes. Most important of all, 499 participants (95.8%) were really expected to acquire more knowledge regarding diabetes. Thus, health education and promotion activities should be developed as soon as possible by policymakers, healthcare providers and educators in the colleges.

In general, this study and the previous studies in other countries, including Oman, Ajman, USA and Mexico^3^ ^10^ ^14^ ^16^ ^17^ all showed that the overall diabetes-related knowledge level was low among the young people, so attempts to reduce the incidence of type 2 diabetes should include young people who are at an impressionable age and can be motivated to make healthy lifestyle modifications.^3^ Meanwhile, college students who were receiving a high education in the colleges had a mean score of diabetes only 59% of the maximum possible total score (ie, 13 points of 22), which was similar to the general population in this country. Recently, a systematic review and meta-analysis of studies published from 1979 to 2012 in China's mainland showed that the pooled estimate for the awareness of diabetes was 45.81% and there was no obvious improvement in the awareness rate from 1998 to 2011 in the general population.^18^ The low awareness rate, independent of one person's level of educational, may be a result of ignoring the propaganda and education about diabetes for a long time in China. Given the total number of people with diabetes has doubled from 20.8 million in 2000 to 42.3 million in 2013 caused by the rapid socioeconomic development and changes in lifestyle,^19^ it is of equal importance to take urgent measures targeting colleges and communities to increase the general public's awareness of diabetes from a public health perspective.

Previous research has shown that 80% of type 2 diabetes cases can be prevented by ameliorating lifestyle, changing to a healthy diet and increasing one's physical activity. However, this study found that only 48.3% of participants recognised that sedentary lifestyles were associated with the increased risk of diabetes. The rate was similar to that reported among university students in Ajman, where 53% of participants did not know that decreased physical activity and bad diet were the major modifiable risk factors for the development of diabetes.^10^ At the same time, a lack of exercise was a serious concern, as only 40% of our study population claimed that they exercised more than 3 days per week. Moreover, even participants who took part in regular exercise did not have a significantly higher score of diabetic knowledge than those who did not exercise. Thus, these results suggested that it is necessary to further clarify the role of physical activity as a preventive measure for diabetes among college students.

The incidence and impact of diabetes-related complications on public health is alarming. Common complications include nephropathy, retinopathy, neuropathy, cardiovascular disease *et al*, which further contribute to a reduced quality of life.^20^ However, the level of knowledge regarding complications of diabetes among college students was low and the rates of awareness of diabetic peripheral neuropathy and eye problems were only 19% and 34%, respectively. The finding was consistent with a similar study conducted in Ajman.^10^ Another study about the knowledge, attitude and practice of final year medical students in Saudi Arabia on the topics of diabetes also suggested a lack of knowledge of diabetic retinopathy.^13^ So diabetes self-management education is required, that is, patients with diabetes should engage in lifestyle changes and should be strengthened among young people to reduce the risk of complications.

The key area of poor knowledge in this study was clinical knowledge about diabetes, including chronic onset (20%), preferred diet and exercise therapy (35.3%) and Hb1Ac as a better monitoring index (11.9%). The low level might be due to the fact that this knowledge was closely related to clinical medicine and could be strengthened by clinical practice. For example, a study among medical students of Ziauddin University has shown that the knowledge about diabetes was greater in the clinical group compared with the preclinical group.^11^ Despite that type 2 diabetes affects the most productive midlife period, it has also started to appear in younger age groups; thus, it is beneficial for college students to master related knowledge as early as possible to prevent, treat and control diabetes in the future. Moreover, college students who know about diabetes can be used as a medium of knowledge to influence diabetic awareness in the people around them, including their relatives and friends or patients with diabetes.

In this study, grade-differences, academic performance-differences and specialty-differences in diabetic knowledge were found. Not surprisingly, students who were at a higher grade had a good academic performance and those in a medical specialty had more knowledge about diabetes compared with other students. Furthermore, this study found that having relatives or friends with diabetes significantly increased the perceived risk of diabetes, which agreed with previous research.^3^ ^10^ ^21^ ^22^ Additionally, gender was significantly associated with the level of knowledge in a univariate analysis. More female than male students had a general knowledge of diabetes, which was consistent with a study conducted among a college population in Ajman^10^ and a study among high school students in Oman.^3^ However, the gender-difference in diabetic knowledge was not significant in the stepwise linear regression analysis because of the collinearity of independent variables. For example, more female students reported having a relative or friends with diabetes (37.5%) compared with men (26.9%). Similarly, the collinearity of the independent variables existed when treating scores of knowledge of risk factors, symptoms, diagnosis and treatment as dependent variables, respectively. For example, 66.7% of medical students were from grades 3 and 4, in contrast to 35.8% of non-medical ones. So in this study, stepwise regression was used to select independent variables, partly avoiding incorrect estimates due to multicollinearity.

Notably, this study showed that the current major sources of information to gain knowledge regarding diabetes were inconsistent with the best mode to convey the information considered by the participants. Although 61.4% and 46.3% of participants received information about diabetes through books and newspapers, and the TV and Internet, respectively, more than 50% of the participants preferred to believe the information provided by health professionals. A study conducted among high school students in Oman also showed similar results in which 61% of the students preferred healthcare staff to provide the information, but the common sources of information were mass media and the school.^3^ This discordance might be due to the fact that false or exaggerated information appeared more often on the TV and the Internet and in newspapers rather than from other sources. Further, the feeling of mistrust of information from the TV, Internet and newspapers resulted in a lack of knowledge about diabetes among college students. Similarly, physicians were also the most highly trusted information source reported by 62.4% of adults in the Health Information National Trends Survey in USA^16^ Therefore, health professionals and their talks and seminars should be proposed as an effective mode to improve diabetes-related knowledge among college students.

Several limitations must be noted in this study. Although the participants were a randomly selected sample, 12% (79/658) declined to participate and 10% (58/579) provided an invalid questionnaire. Participants in this study may have been more health conscious than the students who were unwilling to complete the questionnaire, so the level of knowledge of the populations may have been overestimated in this study. Another limitation was the use of self-reported data and the assumption that participants responded honestly and accurately.

## Conclusion

In conclusion, the results of this study demonstrate that public education about the chronic progress of diabetes and its treatment and control measures, its complications and risk factors, such as unhealthy lifestyle, is a worthy public health goal in college students. Public education, especially among individuls with non-medical specialties, at a low-level grade, having poor academic performance and having no relatives and friends with diabetes, would be extremely beneficial. Future research should assess what the college students' attitudes and practices on diabetes are and whether some targeted educational programmes to increase awareness about type 2 diabetes to prevent and control the occurrence of diabetes are successful among college students.
